# Evaluation of 3D-Printed Connectors in Chair Construction: A Comparative Study with Traditional Mortise-and-Tenon Joints

**DOI:** 10.3390/ma18010201

**Published:** 2025-01-05

**Authors:** Antoniu Nicolau, Marius Nicolae Baba, Camelia Cerbu, Cătălin Cioacă, Luminița-Maria Brenci, Camelia Cosereanu

**Affiliations:** 1 Faculty of Furniture Design and Wood Engineering, Transilvania University of Brasov, B-dul Eroilor, nr. 29, 500036 Brasov, Romania; antoniu.nicolau@unitbv.ro; 2 Faculty of Mechanical Engineering, Transilvania University of Brasov, B-dul Eroilor, nr. 29, 500036 Brasov, Romania; mariusbaba@unitbv.ro (M.N.B.); cerbu@unitbv.ro (C.C.); 3 Department of Management and Military Sciences, “Henri Coanda” Air Force Academy, Str. Mihai Viteazul 160, 500183 Brasov, Romania; catalin.cioaca@afahc.ro

**Keywords:** 3D printed connector, PLA, Fused Filament Fabrication, chair, mortise-and-tenon joint, L-type corner joint, digital image correlation, finite element method

## Abstract

The present paper investigates the possibility of replacing the traditional L-type corner joint used in chair construction with a 3D printed connector, manufactured using the Fused Filament Fabrication (FFF) method and black PLA as filament. The connector was designed to assemble the legs with seat rails and stretchers, and it was tested under diagonal tensile and compression loads. Its performance was compared to that of the traditional mortise-and-tenon joint. Stresses and displacements of the jointed members with connector were analyzed using non-linear Finite Element Method (FEM) analysis. Both connector and mortise-and-tenon joint were employed to build chair prototypes made from beech wood (*Fagus sylvatica* L.). Digital Image Correlation (DIC) method was used to analyze the displacements in the vicinity of the jointed members of the chairs. Seat and backrest static load tests were carried out in order to verify if the chairs withstand standard loading requirements. Results indicated that the 3D printed connector exhibited equivalent mechanical performance as the traditional joint. The recorded displacement values of the chair with 3D-printed connectors were higher than those of the traditional chair reaching 0.6 mm on the X-axis and 1.1 mm on the Y-axis, without any failures under a maximum vertical load of approximately 15 kN applied to the seat. However, it successfully withstood the loads for seating and backrest standard tests, in accordance with EN 1728:2012, without any structural failure. This paper presents a new approach for the chair manufacturing sector, with potential applicability to other types of furniture.

## 1. Introduction

Additive manufacturing (AM), also known as 3D printing technology is a fabrication method where an object is created by successively depositing layers of material [1] based on computer-aided design, and it is used to build prototypes or series products [2]. Before printing, the virtual model is segmented into cross-sectional planes of the designed part, which are then transmitted to the 3D printer. This technology is distinct from subtractive processes, where raw materials are processed to form the final product through techniques like turning, milling, or drilling [1].

After three decades of development, 3D printing technology has emerged as a leading method that does not require any additional processing devices or auxiliary resources. Its ability to produce custom parts on demand has made it increasingly popular over the last decade. 3D printing has evolved into a viable technique for rapid prototyping, mass manufacturing and the production of customized parts [3,4,5].

The Fused Filament Fabrication (FFF) method involves applying material under constant pressure through a nozzle. The extruded material is deposited at a constant rate, solidifying after passing through the nozzle, thereby adhering to the previous material layer [4,6,7]. Complex shapes may require support during the printing process [8]. Additive manufacturing is capable of producing fully functional components using a wide range of materials, including ceramics, metals, and polymers, as well as combinations in hybrid or composite forms [9,10]. Among the diverse polymers available in the market are nylon, polycarbonate, high-density polyethylene, and polystyrene; however, polylactic acid (PLA) is the most commonly used filament due to its average tensile strength of 56.6 MPa, affordability, and suitability for a variety of applications [11]. These materials offer outstanding high thermal resistance, high rigidity and excellent resistance to a wide range of chemicals [2,12,13,14] and properties such as ultraviolet (UV) resistance, bio-compatibility, transparency or hardness. These features make them perfect for industries producing special purpose components [15].

PLA is a thermoplastic aliphatic polyester derived from corn and can be considered as an eco-friendly material. PLA is versatile, and PLA-based composites have been investigated in the literature, including composites with various reinforcements [11].

In the traditional furniture manufacturing industry, designing wooden components is often constrained by the execution of joints between parts [16]. Consequently, the design possibilities are frequently limited to L-type corner joints connecting wooden components, typically involving the joining of no more than two pieces in a single joint [13]. The integration of AM technology, whether independently or combined with traditional methods, allows for the creation of complex and visually appealing geometries that cannot be achieved solely through conventional techniques [17]. Hybrid design is a rapidly growing area of research that provides new hardware and software tools for crafting aesthetically appealing furniture products, enabling creative approaches in furniture design and construction. Generally, hybrid practices aim to expand producers’ creative horizons while overcoming the limitations of traditional furniture making [13]. Moreover, by combining reverse design with additive manufacturing, it becomes possible to quickly conceptualize and produce custom products [18].

Over the last few years, numerous studies [19,20,21,22] have focused on determining the mechanical properties of L-type corner joints in furniture. The concepts of dismountable and modular furniture are becoming increasingly relevant, allowing end-users to engage directly in creating personalized designs [23,24]. Dismountable joints facilitate custom designs with press-fit systems, eliminating the need for adhesives or screws, enhancing fit, and simplifying the production and assembly of parts [25,26]. With FFF technology, these plastic connectors of various sizes and shapes can be produced in a single additive manufacturing process step [12]. The utilization of 3D printing technology reduces waste compared to traditional methods, as the 3D printer applies material only as needed to shape the final design made in software such as *AutoCAD*, *Rhino*, or *SOLIDWORKS* [27].

Various methods are being researched to improve joint solutions for modular furniture elements, replacing traditional ones [28]. Although mortise-and-tenon joints remain prevalent in furniture manufacturing, they are irreplaceable for certain types of constructions, particularly chairs. Chairs are subjected to various direct and indirect loads throughout their lifespan. Different mechanical load types—tensile, compression, bending, shear, and torsion—impact the joints in the chair’s structure. These loads can lead to negative effects, such as bending, cracking, or breaking in the connection elements. Static analysis conducted on the load-carrying capacity of chairs suggests that adding stretchers between legs is a suitable solution for users with a weight exceeding 150 kg [29].

Research involving diagonal compression and tensile testing of L-type corner joints has demonstrated that the mechanical performance of these joints significantly depends on the type of joint [30,31]. Bending moment testing of L-type corner joints connected with 3D printed connectors has been explored in other studies [32,33,34,35,36], and recent research has increasingly concentrated on designing and testing connectors for furniture part junctions, focusing particularly on case furniture [5,24,28,37]. FEM analysis, followed by experiments are utilized to characterize the mechanical performance of the raw material as well as the behavior of the joint in other application fields [36].

This study aims to investigate a specially designed connector for chair construction through testing it in the L-type corner joint under diagonal tensile and compression loads, and comparing it with a traditional mortise-and-tenon joint. Mechanical testing simulation via FEM highlighted the potential damage locations for the newly designed connectors. The research continued with the analysis of the chairs constructed with both traditional mortise-and-tenon joints and connectors for comparative results. Both prototypes underwent tests for seat and backrest strength following EN 1728:2012 [38], and optical analysis assessed 3D deformations in the areas of seat assembly using the DIC method. Both chairs met the standard requirements without structural or joint damage.

The DIC analysis enabled visualization of displacements in the joint area, demonstrating that the chair with connectors withstood a seating load of nearly 15 kN without sustaining damage. The advantages of this chair with 3D printed connectors compared to the traditional model include: disassembly, leading to reduced costs in packaging and transport; transferring assembly operations to the end-user; streamlining the wood-working process by eliminating machining operations for joints and gluing; and reducing the dimensions of seat rails and stretchers, positively impacting solid wood resource utilization. Other advantages of 3D printing in furniture construction over classic manufacturing technology are related to the approach to a new design without restrictions on shape, geometry and joint angle, as well as the use of ecological and biodegradable materials for the additive manufacturing.

## 2. Materials and Methods

Beech wood (*Fagus sylvatica* L.) with a density of 698 kg/m^3^ and a moisture content of 8.5% was used as raw material for the wooden parts of the L-type corner joints and for the chairs, two constructed traditionally with mortise-and-tenon joints and the other two with connectors. The L-type corner joints and the chairs built with the traditional mortise-and-tenon joints serve as a reference. The bonding adhesive used for these structures was the commercial Novobond D2 polyvinyl acetate. However, screws were used to secure the connectors to the chair legs.

**Figure 1:**
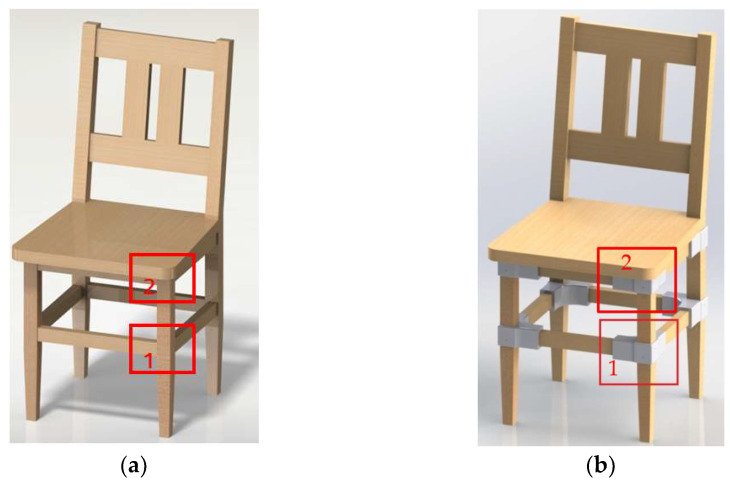
The joints considered in the experimental research: (**a**) Reference chair with traditional joints; (**b**) Proposed chair with 3D printed connectors. (1—leg and stretcher joint; 2—leg and seat rail joint).

The experimental model of L-type corner joint includes three members, one for the leg, with cross-section of 35 mm × 35 mm and length of 50 mm, and two members for the stretchers/seat rails with cross-sections of 35 mm × 22 mm and lengths of 180 mm for the joints with connectors and 222 mm for the reference specimens. The tenon of the reference sample was executed with dimensions (L × l × g) of 12 mm × 13 mm × 8 mm. These joints are marked on the 3D models of the chairs designed for this research in Figure 1a for the reference and in Figure 1b for the proposed chair with connectors.

**Figure 2:**
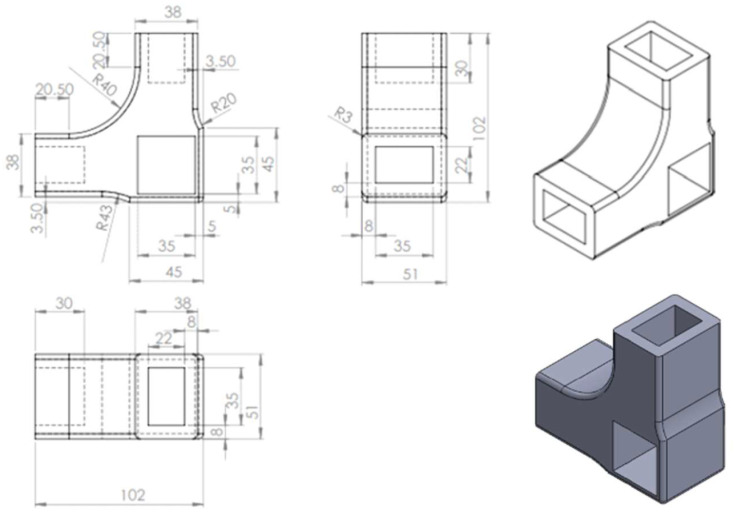
Dimensions and shape of the 3D printed connector.

The connector was 3D printed with the sizes and shape presented in Figure 2.

**Figure 3:**
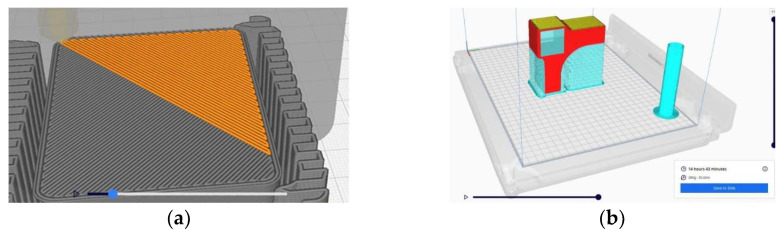
Description of the printing process: (**a**) Adjacent layers deposition; (**b**) Position of the sample on the build platform, displayed by the software.

The software utilized to create the 3D model was *SOLIDWORKS 3D CAD*, version 2016, developed by Dassault Systèmes, France. The (.stl) file of the model was exported to the printer. FFF technology was employed in the additive manufacturing process using the Ultimaker S5 printer (manufactured by UltiMaker, Utrecht, The Netherlands), which has a buildable volume of 330 mm × 240 mm × 300 mm, and operates with its own software, UltiMaker Cura 4.3. The printer deposited filament layers inclined at 45 degrees to the exterior perimeter and at 90 degrees to the previous layer (Figure 3a). The position of the connector on the build platform is displayed in Figure 3b. This position required additional support, which was removed once the connector was fully printed. Previous research has indicated that the horizontal position of parts during the build process tends to be unfavorable for the mechanical resistance of connectors subjected to diagonal compression and tensile loads in a L-type corner joint assembly [33,39].

The filament used for the FFF method was black polylactic acid (PLA) sourced from FORM Futura (Nijmegen, The Netherlands), characterized by a diameter of 2.85 mm, specific gravity of 1.24 g/cm^3^, print temperature range of 190 °C to 225 °C, and a melting temperature around 210 °C, PLA is a bio-plastic, made up of a repeating chain of lactic acid and it is recyclable using conventional methods. Specifically, PLA is a thermoplastic aliphatic polyester derived from corn and can even be composted like other organic materials [11]. With a tensile modulus of 3310 MPa and a flexural modulus of 2364.3 MPa, black PLA demonstrates higher rigidity compared to other filaments, such as white PLA or fiberglass-reinforced PLA, as observed in preliminary experimental research. The selected print parameters were, as follows: print speed of 50 mm/s, print temperature of 250 °C, layer height of 0.2 mm, and 100% fill density. The printing parameters were selected according to the manufacturer recommendations and as result of the preliminary research on this topic [33,39]. Three pieces were printed simultaneously, with a total execution time of 14 h and 43 min.

**Figure 4:**
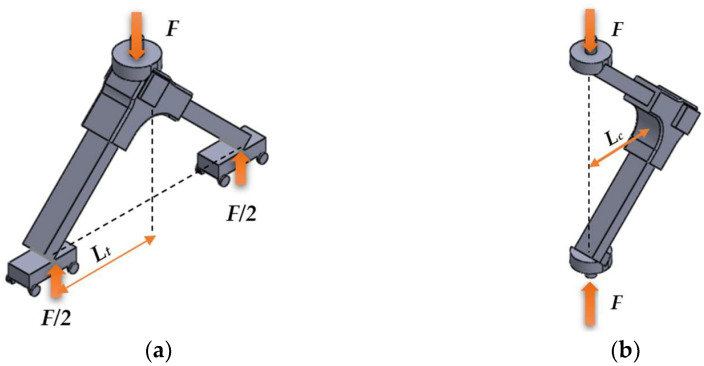
The models of the mechanical tests: (**a**) under tensile load; (**b**) under compression load.

For new models of 3D printed furniture and connectors, it is essential to analyze mechanical properties before bringing the products to market [2]. Following methodologies described in the literature [16,19,20], mechanical tests were conducted on L-type corner joints subjected to diagonal tensile (Figure 4a) and compression (Figure 4b) loads. A Zwick/Roell Z010 universal testing machine (Ulm, Germany) was employed to test five specimens of each category (one group with connectors and the other as the reference group). As depicted in Figure 4a, the tensile force (*F*) tends to open the joint, while the compression force tends to close it.

The bending moments were calculated with Equation (1) for the tensile test and (2) for the compression test.

(1) $$M_{t}=\frac{F}{2}\times L_{t},\;\mathrm{in}\;N\cdot m$$

(2) $$M_{c}=F\times L_{c},\;in\;N\cdot m$$

where *M_t_* and *M_c_* are the moments under tensile and compression loads; *L_t_* and *L_c_* are the moment arms under tensile and compression loads, in m; *F* is the maximum failure load, in N.

**Figure 5:**
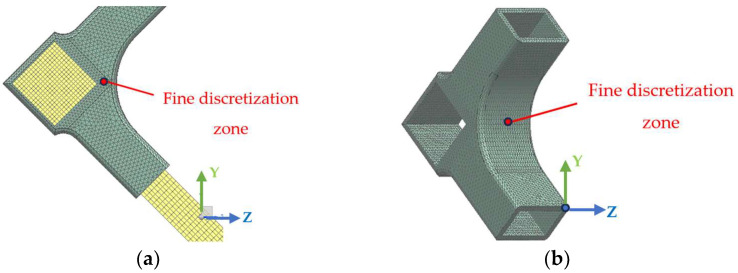
Mesh of the model in FEM analysis: (**a**) detail of the discretization of the L-type corner joint; (**b**) detail of the discretization of the connector.

The models shown in Figure 4 were analyzed using the finite element method (FEM), to simulate how the specimen reacts to applied diagonal tensile and compression forces, resulting in a field of displacements and specific stresses and strains in the connector. The FEM analysis was conducted using the *Simcenter 12.0* simulation software from Siemens PLM Software package, developed by Siemens Industry Software, Brașov, Romania. The initial step involved transferring the (.stl) models from the *SOLIDWORKS 3D CAD* software version 2016to the *Simcenter12.0* for discretization (Figure 5a).

Through discretization, the structure characterized by an infinite number of points is transformed into a simplified model with a finite number of nodes. The discretization was done differently for the solid wood elements and for the connector (Figure 5a). A more complex and finer network with numerous numbers of nodes was applied to some parts of the connector, in order to evaluate better the values of the specific displacements, stresses and strains in the areas of direct contact with the wooden members of the L-type corner joint (Figure 5a,b). The next step was to define rigid connections between the leg segment and the connector. The elastic properties of the beech wood and of the black PLA are presented in Table 1.

**Figure 6:**
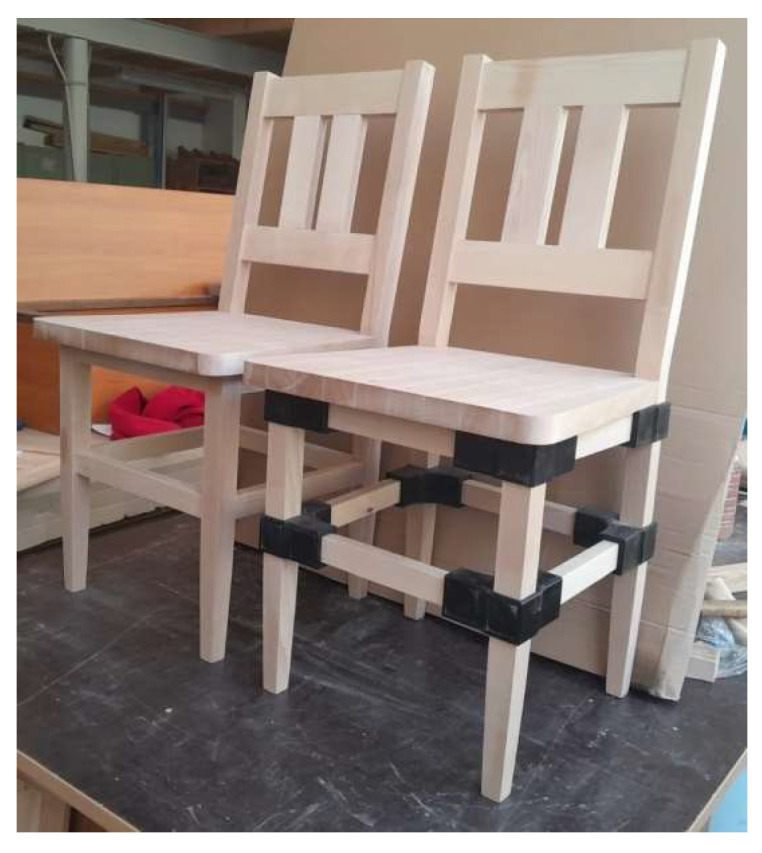
The two types of chairs subjected to final tests (experimental chair with 3D printed connectors on the right, and the reference one on the left).

To evaluate the connector in the final product, four prototypes of chairs were manufactured—two for each type presented in Figure 6. Eight connectors were used to assemble the legs with the seat rails and the four stretchers of the experimental chair. The second type of chair was constructed traditionally with mortise-and-tenon joints and deemed the reference chair, subjected to the same tests as the experimental chair. Both types of prototypes were made from beech wood and had identical component parts, with the exception of the seat rails and the stretchers, which were shorter for the chair with connectors.

The objective of the final tests was to compare the performances of the two chairs— the first built with connectors and the second assembled in the traditional method. The initial test involved an optical analysis of the 3D deformations around the joints using the DIC method, followed by the assessment of the seat and backrest strengths according to EN 1728:2012 standard [38].

**Figure 7:**
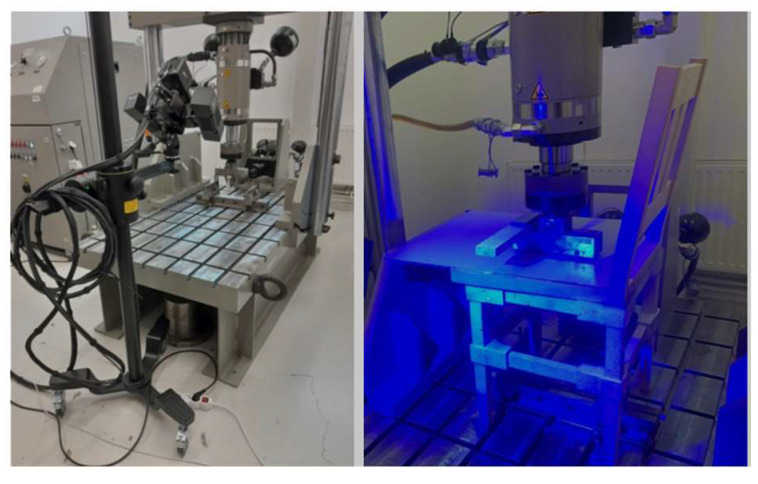
Equipment used for DIC analysis (system for analyzing the behavior of structures in fatigue tests on the left, optical analysis system for 3D deformations on the right).

The equipment for DIC analysis belong to the Laboratory of Mechanical Testing (Research Center of Numerical Simulation, Testing and Mechanics of Composite Materials) in Research & Development Institute of Transilvania University of Brasov (Figure 7), and comprised two main systems:

- A system for analyzing structural behavior in fatigue tests (series 1451, K22305, manufactured by Walter & Bai, Löhningen, Switzerland), featuring actuators capable of moving vertically and horizontally with forces of 100 kN, and 63 kN respectively; piston strokes reaching 100 mm, and DION 7 version 1.6 software for static and dynamic tests.
- The optical analysis system of 3D deformations for materials and components ARAMIS SRX (manufactured by ZEISS GOM Metrology, Braunschweig, Germany), equipped with high-resolution 3D cameras, software, and hardware tailored to determine 3D displacements, strains, strain tensor directions, test point trajectories through digital image correlation methods. It incorporates two optical sensors (CMOS 2 × 12 mega pixels), attaining a resolution of 4096 × 3068 pixels; blue light technology with LED lighting; and GOM Correlate Professional software.

The ARAMIS SRX is a high-resolution 3D camera system capable of full-field and point measurements. It has a potential of capturing 2000 frames per second with two cameras measuring deformation. The blue light technology harnesses a narrowband blue ambient light that interacts during image capture, offering optimal illumination with short exposure times for measurement areas and high accuracy in point-based motion analysis. The DIC method functions by comparing digital images of the tested piece at various deformation stages, thus measuring displacements along the axes.

The monitored surfaces of the tested chairs were painted black and white to create a grid of black points (markers) that visualize the deformations. The GTM force transducer with a nominal load of 100 kN manufactured by GTM—GLASSMANN Testing and Metrology, Bickenbach from Germany was utilized for compression loading. The set parameters were, as follows:

- The vertical movement of the force transducer was fixed at 5 mm;
- Travel speed: 0.02 mm/s;
- Fixed frame rate of 2 Hz, resulting in a total of 400 frames.

**Figure 8:**
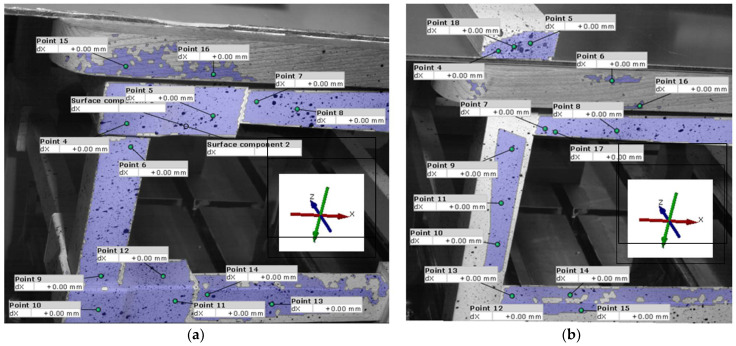
The selected points to follow the displacements on the horizontal and vertical axes: (**a**) For the chair with connector; (**b**) For the reference chair.

The value of the load force on the seats will be determined once the final vertical movement of 5 mm is attained. In the jointing area between the legs and the seat rail and stretcher (monitored surfaces), 16 points were selected for the chair with connectors (Figure 8a), while 18 points were chosen for the reference chair (Figure 8b) to analyze their displacements on the X and Y axes.

The second set of chairs (one with connectors and one as a reference) were tested on a furniture test rig from Hegewald & Peschke Meß- und Prüftechnik GmbH (Nossen, Germany), situated in the Design, Prototyping and Testing Laboratory (Cluj Innovation Park, Regional Center of Excellence for Creative Industries, Cluj-Napoca, Romania), which is certified by the National Accreditation Organization (RENAR). The test rig comprises two axes with 2.5 kN force cells each and is capable of performing cyclic durability tests, fatigue resistance tests on furniture components, and finished furniture products, operated by SIMATIC HMI visualization software from Siemens AG (Munich, Germany). The chairs underwent testing according to the [38] standard for static loads, specifically:

- Seat static load of 1300 N and back static load test of 430 N, (test 6.4);
- Leg forward static load test (test 6.15), by applying a seat vertical load of 1000 N and a horizontal force of 400 N centrally to the rear of the seat, directed forward;
- Leg sideways static load test (test 6.16), by applying a seat vertical load of 1000 N and a horizontal force of 300 N centrally to the unrestrained side of the seat, at seat level, in a direction towards the restrained feet.

**Figure 9:**
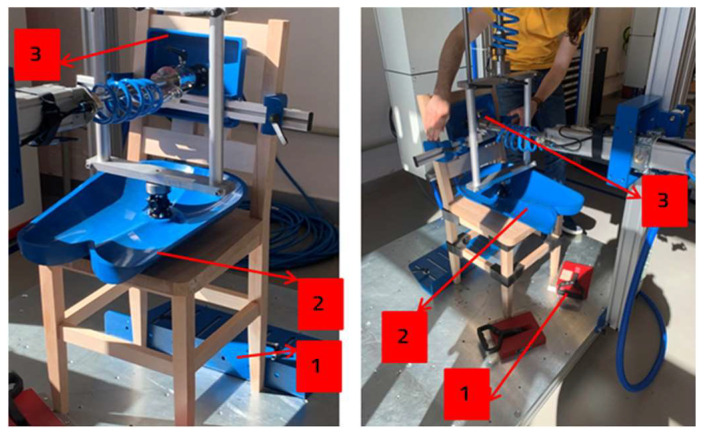
Chairs preparation on the test rig (reference chair on the left and chair with connectors on the right).

Tests were carried out for 10 cycles, following several stages: positioning the chairs on the rig and attaching the locking devices (Figure 9—position 1), setup of the pressing devices for the seat (Figure 9—position 2) and backrest (Figure 9—position 3).

The test was conducted to determine whether the chair with connectors meets the minimum strength requirements under the same conditions as a traditionally manufactured chair. This method has also been applied by other researchers in their study of 3D-printed connectors designed for chairs [34].

## 3. Results and Discussion

### 3.1. FEM Analysis

**Figure 10:**
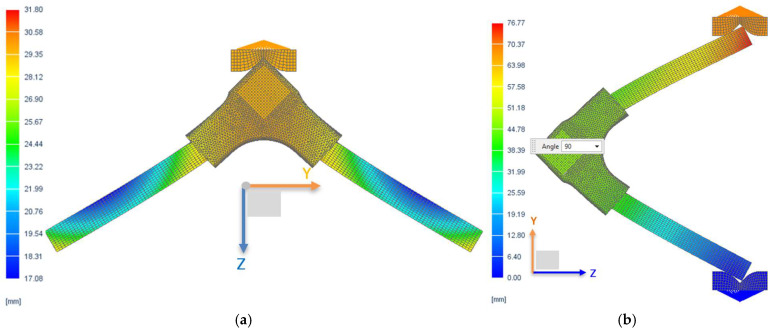
Field of displacements: (**a**) Simulation under the tensile load; (**b**) Simulation under the compression load.

**Figure 11:**
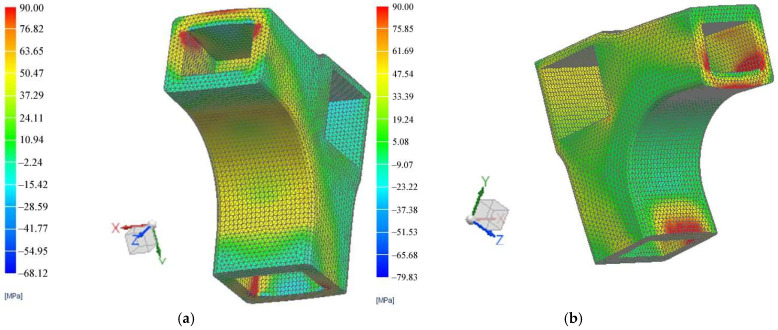
Field of stresses: (**a**) Simulation under the tensile load; (**b**) Simulation under compression load.

**Figure 12:**
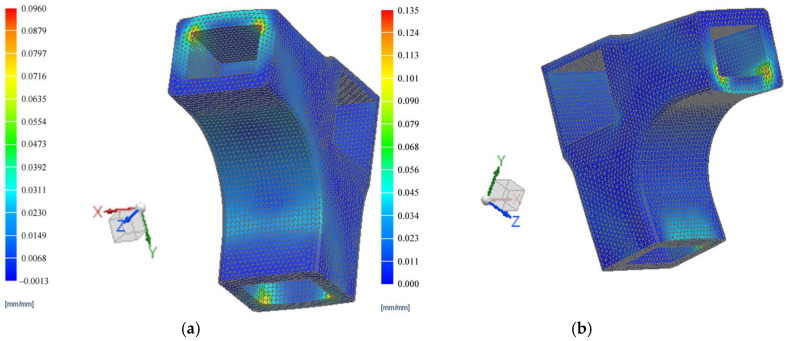
Field of strains: (**a**) Simulation under tensile load; (**b**) Simulation under compression load.

The displacement fields of the L-type corner joint systems subjected both to compression and tensile loads, as well as the strain and stresses in the connector, were delineated through a non-linear FEM analysis. The results are illustrated in Figure 10 for displacements, in Figure 11 for stresses, and in Figure 12 for strains.

Figure 10 displays the deformed shapes of the L-type corner joints corresponding to displacements of 31.8 mm under tensile load and 76.77 mm under compression load, respectively. Deformed meshed geometry (as seen in Figure 10b) of the joint under compression load, accompanied by the loss of its initial position was highlighted also by [36], where numerical simulation was included. The maximum displacements occurred in the connector under tensile load (Figure 10a) at the upper section contacting the pressing device of the testing machine. Significant displacements (around 28 mm) are also indicated at the contact points between the rails and connector, indicating a tendency for the rail to detach from the connector. This tendency influences stress distribution in the connector’s opposite corners and the corresponding outer face of the connector, where stress values reached approximately 90 MPa (Figure 11a). These areas are likely to fracture during experimental testing.

Figure 11b illustrates the maximum stress field in the connector under compression load. The most vulnerable surfaces are the interior corners in contact with the stretchers (or seat rails) that correspond to the connector’s concave surface. The red color on the concave area indicates a stress value of 90 MPa, which is also susceptible to damage the connector during the mechanical testing.

Figure 12 shows the strain fields in the connector resulting from the FEM simulation of the diagonal tensile (a) and compression (b) tests applied to the L-type corner joints. The same corners with maximum stress values also exhibited the highest strain values, reaching 0.096 mm/mm under tensile load and 0.135 mm/mm under compression.

Similar simulations were conducted by other researchers [34] for 3D printed connectors manufactured using fused deposition modelling 3D printers with ABS filament. Their model simulated a compression test, where the force was applied at the end of the horizontal rail while the vertical rail was clamped. Maximum stresses of 26.9 MPa were recorded at the corners of the connectors with wall thickness of 5 mm, at the contact with wooden rails, where failures ultimately occurred after testing. The higher stresses of 90 MPa observed after FEM simulation in this study can be at-tributed to the thicker walls of the newly designed connector, measuring 8 mm, which demand greater pressing forces for deformation.

### 3.2. Mechanical Testing

**Figure 13:**
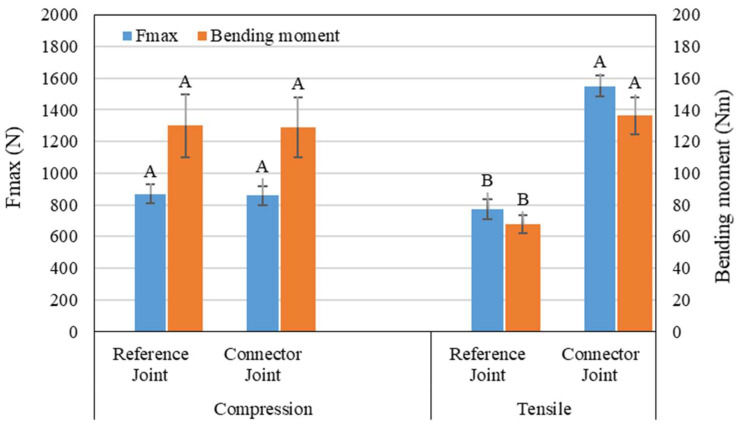
Results of the mechanical tests.

The comparative graphs in Figure 13 demonstrate that the L-type corner connector joint performed better than the reference mortise-and-tenon joint assembly.

An initial observation from Figure 13 is that the bending moment under compression load is greater than that under tensile load for the mortise-and-tenon joint, consistent with trends reported by other researchers for wooden-based panels joints [16]. Testing mortise-and-tenon L-type corner joints composed of beech wood [19], the bending moment under compression load was nearly twice that of the present work, while under tensile load, it was five times greater than the values resulting from the reference samples. These discrepancies can be explained by differences in specimen cross-sectional area and dimensions, corresponding moment arms, tenon sizes, and adhesive characteristics: larger cross-sections and tenon dimensions yield higher bending moments.

In the case of using the connectors, higher forces were recorded under tensile loads in comparison to compression loads (Figure 13). This trend has been noted by other researchers [20,32] investigating L-type corner joints made from beech wood, medium density fiberboard, and particleboard using various 3D printed fasteners designed for case furniture assembly. However, these types of joints typically demonstrate much lower bending moments compared to those recorded in the current study—approximately 12.3 Nm under tensile load and 8.6 Nm under compression load for assemblies made from beech wood [32] and 44.16 Nm under compression load for plywood joints [21]. Additionally, the bending moment values for L-type joints between two components made from glued HDF panels with a cross-section of 40 mm × 40 mm were roughly half of those obtained in this research [23], where 3D printed connectors made of acrylonitrile butadiene styrene (ABS) were used for the joints.

When comparing the bending moment capacity values determined in this study against those produced by other researchers [22] for similar samples constructed from beech wood and assembled with beech Domino dowels, consistency is evident in results. Other mortise-and-tenon corner-joints made of beech wood also exhibited similar bending moment values under compression when compared with the current study; however, higher tensile load bending moment values were recorded [31].

From the above results, one initial conclusion emerges: the values of bending moments under diagonal tensile and compression rely on both the material of the specimens and the joint type. For beech wood, the mortise-and-tenon joint generally exhibited higher values than other joint types [19,31], and tenon size significantly influences the tensile bending moment value: larger tenon sizes yield higher bending moment results. This conclusion applies to 3D printed connectors and fasteners as well. In this instance, the shape, material of the fastener, and the applied method collectively impact the joint’s strength [27]. For example, dowel pins produced using the FFF method with PLA filament achieved mechanical performance comparable to wooden dowels in L-type corner joints con-structed from beech wood [26], highlighting PLA’s suitability as a replacement for traditional wooden joints.

The statistical analysis for the data presented in Figure 13 included the calculation of the standard deviation using *Microsoft Excel* to establish a 95% confidence interval and a significance level of 0.05 (*p* < 0.05). A two-sample *t*-test was conducted using the *Minitab* software package, version 19.2020.1 to evaluate whether the mean tensile and compressive strengths were significantly influenced by the implementation of the connector joint in comparison to the reference case. The *p*-value > 0.9 indicates no significant difference between the connector and reference joints for the maximum force and bending moments under compression. The *p*-value < 9 × 10^−5^, much smaller than the standard significance level (0.05), indicates a statistically significant difference in bending moments under tensile conditions and demonstrates a higher tensile force capacity of the connector joint compared to the reference joint. According to the statistical results, the connector joint shows superior performance in tensile conditions, making it a better choice for applications involving significant tensile stress. For compressive conditions, there is no meaningful difference between the two joint types, suggesting that either joint could be used interchangeably for such applications.

**Figure 14:**
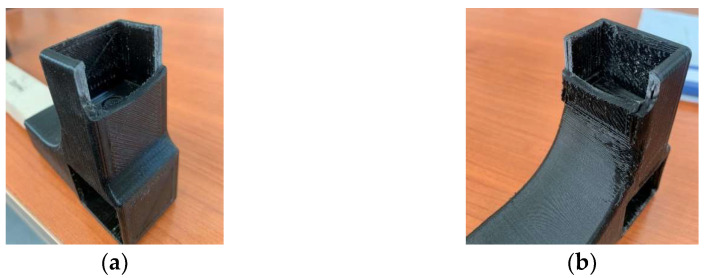
Connector failure: (**a**) Under tensile load; (**b**) Under compression load.

FEM analysis serves as an important approach to simulate joint behavior under bending loads, evaluating displacement magnitudes, stresses, strains, and identifying vulnerable areas of the joint [20,23,28,32,34]. Accordingly, in the present case, the material failure occurred exclusively at the connector within the joint area adjoining the rails, as depicted by FEM analysis (Figure 14), leaving no cracks or other damages on the wooden parts.

From comparisons made, a consistent correlation appears between FEM simulation analyses and real mechanical testing outcomes regarding sensitive areas subjected to maximum stresses and strains, as well as system displacements. Experimental mechanical testing of the L-type corner joint with the connector indicated a maximum displacement value of around 25 mm for diagonal tensile loads and 60 mm for diagonal compression, with maximum forces of 1070 N and 719 N, respectively. These values are closely aligned with those highlighted by the FEM analysis

### 3.3. Optical Analysis of Displacements by DIC Method

**Figure 15:**
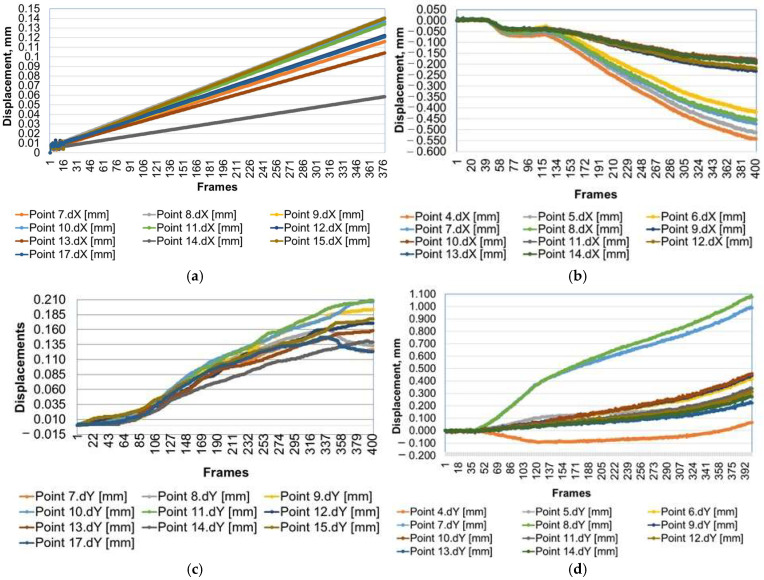
Displacements along X and Y axis, as resulted from DIC analysis: (**a**) points of reference chair along X axis; (**b**) points of chair with connectors along X axis; (**c**) points of reference chair along Y axis; (**d**) points of chair with connectors along Y axis.

To visualize the continuous displacements of the selected points, images captured by the two cameras were downloaded every 50th frame, resulting in a total of 9 images for each set of displacements on the X and Y axes for the 16 and 18 selected points, respectively. Graphical representation of the displacements of points in the jointing area is shown in Figure 15 for both the chair with connectors and the reference chair with traditional joints.

As can be observed in Figure 15, the behaviors of the two chairs differ under the pressing load applied at the center of the seat, until a vertical movement of 5 mm is reached. Initially, the displacements of the reference chair at the selected points are more homogeneous compared to those observed with the chair equipped with connectors. Furthermore, displacement values are higher in the chairs with connectors than in the reference chair, measuring 0.600 mm compared to 0.150 mm on the X axis and 1.100 mm versus 0.210 mm on the Y axis, indicating that the mortise-and-tenon joint exhibits increased rigidity relative to the connector joint. Following the displacements of the points selected on the reference chair, the ones selected on the leg (points 9, 10 and 11) recorded the highest displacements in both the X and Y axes. In contrast, points 7, 13, and 14 located near the mortise-and-tenon joints on the stretcher and seat rail displayed the lowest displacement values, evidencing the high rigidity of these joints (Figure 15a,c).

Conversely, for the chair with connectors, the displacement of points along the X axis occurred oppositely compared to the reference chair, yielding negative values in this instance (Figure 15b). The highest displacement values were noted for points situated on the connector of the seat rail and the seat rail adjacent to this connector (points 4–8), whereas the lowest values originated from points aligned on the connector of the stretcher (points 9–12). This phenomenon can be explained by the tendency of the seat rail to bend under the compression load applied at the chair seat, causing the connector to rise along the Y axis while simultaneously pushing the leg outward, signaling it could disengage from the joint. This action is substantiated by the graph in Figure 15d, which illustrates the negative displacement of point 4 situated on the connector, alongside the peaks in displacements for points 7 and 8 located on the seat rail. In this case, the connector joint may be classified as elastic, allowing movement within the joint system.

**Figure 16:**
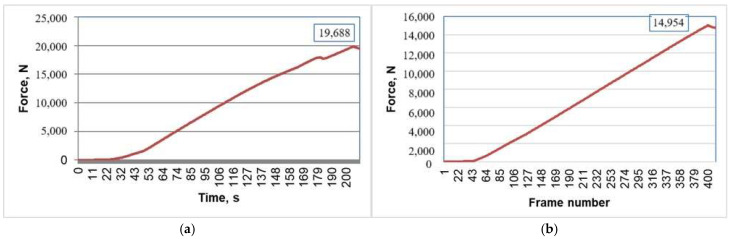
Load sizes corresponding to the 5 mm travel stroke during DIC analysis: (**a**) For the reference chair; (**b**) For the chair with connectors.

Upon completing the optical analysis through the DIC method, both the reference chair and the chair with connectors were able to withstand loads of 19.69 kN (Figure 16a) and 14.95 kN (Figure 16b), respectively, without damaging the wooden components or connectors. As noted in another study [29], the inclusion of stretchers significantly increases of the chairs’ load-bearing capacity. The presence of the four stretchers within the designed chair contributed to the high values of the loads recorded at the end of the test.

Another example can be found in the study [28], where a chair seat was subjected to bending test. 3D printed connectors made from Polyethylene Terephthalate (PETG) using FDM fabrication method were utilized to construct a chair capable of withstanding loads up to 7300 N. However, its structure was relatively simple, without stretchers.

The DIC optical displacement analysis results presented in this section indicate that the 3D printed connectors can effectively replace traditional mortise-and-tenon joints in chair structures while meeting equivalent performance standards, as evidenced by a load-carrying capacity exceeding 1495 kg, which far surpasses functional requirements. Future research could explore optimizing the chair and connector structures to enhance mechanical strength regarding load-carrying capacity and economic efficiency.

### 3.4. Static Load Tests

Static load tests for the seat and back were conducted first. The seat sustained a load of 1300 N, while the backrest supported a load of 430 N, both without damaging the wooden parts or connectors. Subsequently, a vertical seat load of 1000 N, accompanied by a horizontal force of 400 N, was centrally applied to the rear part of the seat, while a vertical seat load of 1000 N and a horizontal force of 300 N were applied centrally to the side of the seat, towards the restrained feet. Both chairs held the applied loads without failures or ruptures. It is crucial to note that the test rig utilized for this research is homologated, and the testing laboratory holds accreditation. As expected, the results of the performed tests revealed that the joint with connector offers the chair the resistance required by the standards in force. No difference was noted during and after the test between the strength of the chair with traditional joints and the chair with the 3D printed connectors.

Another attempt at using 3D printed connectors for chair construction was documented in [34]. In this instance, connectors were fabricated using FDM technology with ABS filament. The static load test applied to the chair’s seat and backrest in accordance with [38] resulted in the backrest failing under load along a 3D printed layer at the connector.

This subject remains open to discovering the best solutions regarding materials and additive manufacturing methods that align with optimal design and economic efficiency.

## 4. Conclusions

- 3D printed connectors present a viable solution for chair construction.
- Joints with 3D-printed connectors made of PLA filament using the FFF additive manufacturing method exhibited greater strength under diagonal compression and tensile loads compared to traditional wooden mortise-and-tenon joints made from beech wood.
- FEM analysis of diagonal compression and tensile loads applied to L-type corner joints with connectors identified the susceptible zones of the connectors under maximum strain and stress.
- Experimental testing have shown that the connectors failed at the zones revealed by FEM analysis under maximum forces without damaging the wooden parts. Optical displacement analysis using the DIC method had as result higher values of the displacements for the chair with connectors compared to the reference chair, reaching values of 0.6 mm along the X axis and 1.1 mm along the Y axis under maximum vertical load on the seat of about 15 kN without failures of the chair structure.
- The reference chair exhibited uniform and consisten displacement patterns near the joints, highlighting the rigidity of the structure.
- The chair with connectors displayed varying displacement trends, attributed to the connector elasticity, which allowed movement between the wooden parts and the connectors without inflicting damage.
- Compressive loads applied to the chair seats achieved notable values of 19.6 kN for the reference chair and approximately 15 kN for the chair with connectors, without compromising their structural integrity. Both chairs withstood loads of 1300 N for the seat and 400 N for the backrest, meeting the mandatory requirements set forth by relevant standards.
- The chair with 3D-printed connectors is a reliable alternative to traditionally manufactured chair and a sustainable solution: it is an ecological option, ensures the product’s disassembly, and allows for replacement to extend chair lifespan.
- The estimated cost of € 9.6 per connector can be reduced by further research, which could aim at optimizing both the chair and connector designs to align the mechanical strength of the chair with its load-carrying capacity while considering economic benefits.

## Figures and Tables

**Table 1:** Elastic properties of the materials used for FEM analysis *.

Material	Young’s Modulus, in MPa			Shear Modulus, in MPa			Poisson’s Ratio					
	*E_L_*	*E_R_*	*E_T_*	*G_LR_*	*G_LT_*	*G_RT_*	*ν_TR_*	*ν_RT_*	*ν_LT_*	*ν_TL_*	*ν_RL_*	*ν_LR_*
Wood	14,000	2280	1160	1970	950	467	0.36	0.75	0.51	0.044	0.073	0.45
PLA	3149			1287			0.36					

* Note: *L* indicates the longitudinal section; *T* denotes the transverse (crosscut) section, and *R* represents the radial section.

## Data Availability

Data are contained within the article.
